# ‘Spatio-market practices’: conceptualising the always spatial dimensions of market making practices

**DOI:** 10.1007/s13162-021-00203-1

**Published:** 2021-07-10

**Authors:** Torik Holmes, Josi Fernandes, Teea Palo

**Affiliations:** 1 Cardiff School of Geography and Planning, Cardiff University, Cardiff, CF10 3AT UK; 2 Department of Marketing, Lancaster University Management School, Bailrigg, Lancaster, LA1 4YX UK; 3 University of Edinburgh Business School, 29 Buccleuch Pl, Edinburgh, EH8 9JS UK

**Keywords:** Market making, Spatial theory, Spatio-market practices, Casino gambling

## Abstract

Socio-material conceptualisations of markets suggest that they are spatial formations. Yet, the everyday practical and spatial dimensions of market making have received little explicit attention. We thus introduce the concept of spatio-market practices, drawing on key ideas in market studies and spatial theory. We argue that examining spatio-market practices (and thus the spatial dimensions of markets) promises to provide fresh insight regarding what it takes to realise markets, their uneven distribution, and what and whom markets are (and are not) designed to serve. To demonstrate what the concept calls for, supports and promises, we take Humphreys’ (2010) influential paper as a starting point and draw on other secondary sources in order to articulate an alternative and spatially-oriented account of the growth and legitimacy of the American casino gambling market. This paper, in turn, contributes a subtle and yet incisive shift in thinking, which supports a more explicit means of exploring markets as spatial formations.

## Introduction

The lack of attention of the marketing discipline to markets and their making has been widely discussed (Araujo et al., 2010; Mele et al., 2015; Vargo & Lusch, 2016; Venkatesh et al., 2014). While views on product markets as abstract conceptions have dominated much of the marketing literature (Day, 1981; Rosa et al., 1999), markets have increasingly been conceptualised and approached as socio-material formations (Araujo, 2007; Kjellberg & Helgesson, 2007; MacKenzie, 2009). From this standpoint, markets are made through the material relationships between multiple human and non-human actors and the practices they enact, configure and perform (Araujo, 2007; Araujo et al., 2008; Callon & Law, 2005; Callon & Muniesa, 2005; Kjellberg & Helgesson, 2007).

The emphasis placed on the necessary performance of markets and practice comes with an implicit suggestion that markets are spatial formations. This is because markets and practices are understood to take place *in,* while also being distributed *across,* physical and representational spaces (Cochoy, 2008, 2009; Finch & Geiger, 2010). Finch and Geiger (2010), for instance, write that “relationships between producers and consumers, buyers and sellers, are enacted or performed in market spaces” (p. 238). Similarly, Kjellberg and Helgesson (2006) posit that conflicting practices can endure only if they are “[separated] in time and space” (p. 850). And, Peñaloza and Venkatesh (2006, p. 147) write that “markets… take on distinct discursive forms and material practices across various social contexts and over time.” These quite ambiguous treatments of space as a neutral container and backdrop leave more to be said about the spatial dimensions of market practices. Indeed, left unaddressed are questions concerning the production of market spaces through practice, what this takes, and related consequences. These questions are particularly pertinent, as work focused more explicitly on the spatial dimensions of marketing and economic geography have tended (as we will show below) to draw on established spatial concepts, as opposed to explicitly theorising the everyday spatiality of market making and thus market practices.

We therefore take a different approach to that taken in market-oriented work to date. Specifically, we introduce the concept ‘spatio-market practices,’ drawing on ideas generated in market studies and the spatial theories of Lefebvre (1991) and Harvey (1973, 2006). This concept is premised on three propositions: 1) market actors are always engaged in and responding to the production of, exchange in, and consumption of tangible and imagined market contexts; 2) the production, exchange and consumption of such contexts are emanations of the performance and wider ordering of market practices; 3) and, such performances and the wider ordering of markets and thus patterns of production, exchange and consumption, hinge on the mobilisation of various conceptualisations of space. By introducing this concept, we make the case that questions concerning the spatiality of markets−such as “is 'the market' a space? A place? A region? All of these things? Or something else?” (Alvarez León et al., 2018, p. 214)−should be approached in reference to the performance and dynamics of specific spatio-market practices. In this respect, our conceptualisation of spatio-market practices is not diametrically opposed to established work. Instead, it complements ongoing discussions, contributing a subtle and yet incisive shift in thinking that supports a particular means of exploring the spatial dimensions of market making.

The article is structured in the following way. In the coming section, we discuss in more detail how space has been treated in market-oriented research, including marketing and economic geography. In the subsequent sections and drawing on key contributions in market studies and spatial theory, we articulate the idea of spatio-market practices. To demonstrate what the concept calls for, supports and promises, in the penultimate section we take Humphreys’ (2010) influential paper as a starting point and draw on other secondary sources to articulate an alternative and spatially sensitive account of the growth and legitimacy of the American casino gambling market. In the final section, we conclude with a discussion of what our conceptualisation of spatio-market practices calls for in future research and what it offers to scholars interested in markets, marketing and market making.

## Marketing, market geographies and space

Marketing scholarship and in particular consumer research has drawn on spatial concepts in an effort to understand “how consumers consume across a gamut of social spaces” (Arnould & Thompson, 2005, p. 875). Studies have, for example, scrutinised the physical environments in which market exchanges take place by examining retail shelf space allocations and store presence (Hübner & Kuhn, 2012; van Herpen et al., 2012; Van Nierop et al., 2008). In other cases, retail atmospherics and the use of sensory marketing have formed key concerns (Shankar et al., 2011; Turley & Milliman, 2000). Echoing this interest in affect, within the services marketing domain, the notion of ‘servicescape’ has been developed (Bitner, 1992). This concept refers to the firm-controlled elements in the physical service environment that can enhance (or control) customer and employee actions. Important elements include materials, spatial layout and functionality, ambient conditions, and signs and symbols (Aubert-Gamet & Cova, 1999; Bitner, 1992; Harris & Ezeh, 2008; Houliez, 2007; Nilsson & Ballantyne, 2014; Tombs & McColl-Kennedy, 2003). In work concerned with servicescapes, space is typically treated as a canvas, populated with material elements (e.g., equipment and furnishings). As O’Leary et al. (2019) explain, space is treated in absolute terms, as something ‘out there.’ By implication, the dynamics of market making are reduced to objective encounters *in* space. As a result, market practices are somewhat detached from the production of spaces and vice versa the uneven production of spaces from the performance of market practices.

Within a more recent corpus of work on markets within marketing, existing spatial concepts have been deployed to explain market dynamics (Castilhos & Dolbec, 2018; Castilhos et al., 2017; Chatzidakis et al., 2018; Vicdan & Hong, 2018). Utilising Jessop et al.’s (2008) 'TPSN analytical framework,' Castilhos et al. (2017) review market-based studies to draw out the key insights articulated in works that use spatial concepts such as territory (T), place (P), scale (S), and network (N). Going a step further, Castilhos and Dolbec (2018) conceptualise and distinguish public and market spaces, defining the latter as “owned and governed by one or multiple market actors” (p. 158). Examples of such market spaces include shops, shopping centres, and entertainment venues, all of which are conceptualised as bounded spatial arrangements, imagined and designed to make specific consumer practices more likely to happen. Warnaby and Medway (2013) likewise emphasise how market making involves the orchestration of narratives or representations that are inscribed in spaces, focusing specifically on the role of ‘place’ in place marketing. Similarly, Chatzidakis et al. (2012), Lloveras et al. (2018) and Roux et al. (2018) extend Foucault’s (1986) concept of heterotopia as part of considering the social construction and functioning of sited markets. As Roux et al. (2018, p. 219) write, citing Foucault (1986, p. 24), heterotopias are ‘‘places that do exist and that are formed in the very founding of society.” While this body of work demonstrates how the crafting of market spaces forms an integral part of production and consumption processes in and across different sites, the inextirpable spatial dimensions and dynamics of everyday market making and marketing practices remain somewhat implicit, as opposed to explicit.

The case is similar in many contributions to economic geography. Contributions in this field have focused on ‘the geographies of marketization’ to explain how representations and ideal models of markets are enacted across space (Alvarez León et al., 2018; Berndt & Boeckler, 2009, 2011, 2012; Boeckler & Berndt, 2013). In this tradition, Alvarez León et al. (2018) specifically ask, in the introduction to a recent special issue: “What is gained by a conceptualization of markets that foregrounds their spatial constitution?” and “how can this approach to markets contribute to the development of a more robust geographic political economy?” (p. 211). Though pertinent questions, contributions to the issue mainly extend existing spatial concepts to discuss the uneven production of neo-liberal and capitalist geographies (Ashton & Christophers, 2018; Hall, 2018). The concern with ‘large-scale’ socio-political and economic relations and structures, mean that the spatial dimensions of everyday market activities remain largely mute (Christophers, 2014a, 2014b).

By contrast, within ‘market studies,’ the everyday performance of markets is a central concern (Araujo, 2007; Araujo et al., 2008; Kjellberg & Helgesson, 2007). This follows a broader turn across social theory toward conceptualizing and studying socio-materiality (Bennett & Joyce, 2013; Mukerji, 2015). The roots of this turn and of market studies lie in actor-network theory (ANT) (Latour, 2005) inspired approaches to theorise markets, first in Economic Sociology and Science and Technology Studies (STS), and then in Management, Marketing, and more recently in Organisation Studies (Çalışkan & Callon, 2010; Callon, 2009; Cochoy et al., 2016; Palo et al., 2018).

The spatiality of markets is a recurrent and yet also relatively ambiguous feature of contributions within market studies which follow the socio-material tradition. Araujo (2007, p. 215) writes, for example, that markets are “dynamic… learning spaces whereby supply and demand are continuously being reshaped.” Similarly, Kjellberg and Helgesson (2006) expand ideas around the simultaneous existence of a multiplicity of markets, arguing that in order to settle conflicts, market practices must be separated “in time and space” (p. 850). Likewise, Finch and Geiger (2010) argue that it is through the performance of practices that actors “help disentangle goods and services in a market space” (p. 237). Looking at the bounding of socio-material arrangements, Stigzelius et al., (2018, p. 347) further conceptualise the kitchen as a market-consumption junction, “where multiple concerned actors in markets and consumption come to shape, and get shaped by […] practices in the kitchen.” Here, the kitchen is conceptualised as “an abstract space of political negotiations” and “a concrete place where different actors and artefacts come together” (Stigzelius et al., 2018, p. 348). Together, these studies suggest that market making is very much a spatial affair, linked with the performance and ordering of multiple connected and situated socio-material market practices. Yet, they leave more to be said about the spatial dimensions of market practices and in particular how conceptions of space are crucial to the performance of markets and spaces of production, exchange and consumption.

Though socio-material contributions, and in particular those linked with market studies, leave more to be said about the everyday spatial dimensions of market making, the concern with the performativity of markets through ‘market practices’ represents a useful conceptual starting point to begin thinking about such dynamics. Indeed, it sets a foundation for thinking about and exploring the spaces market actors make and the spaces their actions are shaped by. Taking this step offers an opportunity to go beyond treating space as a neutral backdrop or deploying established analytical concepts, such as site, place, territory and scale, in a way that suggests they are a precursor to practices rather than their product. To move in this direction, we turn to pre-existing contributions that explicitly concern the ordering and performance of market practices.

## Market practices

A turn towards the conceptualisation and study of ‘practices’ has unfolded in a corpus of work falling under the heading of ‘market studies’ (Araujo, 2007; Araujo et al., 2008; Kjellberg & Helgesson, 2007). This turn builds on the premise that markets have to be performed by market actors and that markets do not exist a priori (Kjellberg & Helgesson, 2006; MacKenzie et al., 2007; Mason et al., 2015).

Araujo et al. (2008, p. 8) broadly define market practices as “the bundles of practices including material arrangements that contribute to perform markets.” Additionally, Araujo (2007, p. 218) writes that “marketing as a practice is deeply rooted in specific market contexts, spatially distributed, and dependent on complex forms of coordination amongst different actors and heterogeneous bodies of expertise.” Practices can thus be understood to comprise socio-material formations, which are heterogeneous in nature, connected, distributed across space, and contextual. By examining the necessary performance of markets through the lens of practice, scholars have problematized the idea of the ‘market,’ showing how multiple practices, connected materials, skills, ideas, aims and imaginaries, underpin instances of exchange. Applying the practice lens has, in turn, shone fresh light on the dynamics of market contestation, the significance of embedded value-systems, and what and whom markets are designed to serve.

The contextual and distributed nature of practices implies that they have spatial dimensions, which are crucial to the performance and ordering of markets. This implication is echoed in Kjellberg and Helgesson’s (2007) analytical distinction of three types of practices−and no one can avoid trial by spaceexchange practices; representational practices; and normalizing practices. “Exchange practices… refer to the concrete activities related to the consummation of individual economic exchanges” (Kjellberg & Helgesson, 2007, p. 142). These include those to do with the specification and presentation of products, price negotiations, and delivery terms and processes. As the authors explain, these activities (and others alike) are those that “contribute to temporarily stabilize certain conditions” to make sited instances of exchange possible (Kjellberg & Helgesson, 2007, p. 142). By contrast, “representational practices include activities that contribute to depict markets and/or how they work” (Kjellberg & Helgesson, 2007, p. 143). Representational work is crucial to making abstract products and markets meaningful. This work is particularly “necessary to bridge temporal and spatial distances between individual exchanges,” making markets and products cogent discursive entities that can be traded and strategized (Kjellberg & Helgesson, 2007, p. 143). Normalizing practices are, instead, those aimed at directing how a market ‘should’ work (Kjellberg & Helgesson, 2007). Such practices include efforts to bring about “market reforms…, [the specification of] general rules of competition and marketing…, and activities related to strategic planning and [the] establishment of objectives by individual market actors” (Kjellberg & Helgesson, 2007, p. 143).

Based on these conceptual distinctions, it is clear that market practices have spatial dimensions. Though, the nature of these remains relatively ambiguous. Broadly speaking, it is clear, for example, that market practices, embedded actors and representational meanings, are unevenly distributed across market space(s). It is also clear that market practices involve imagining and designing market sites that have physical and representational qualities. Likewise, it is clear that market practices take place ‘in’ market sites and in reference to value-laden, normative and contextual expectations. Yet, the spatial dimensions of the performance of markets more broadly have not formed an explicit conceptual concern. This is to say that the making of market contexts and the spatial distribution of practices have not been approached first and foremost as spatial affairs, which further depend on the necessary mobilisation of various conceptualisations of space. To think through and help open up the spatiality of market practices, we introduce the concept ‘spatiomarket practices.’ This spatially sensitising concept sets the ground to derive fresh insight regarding what it takes to realise market contexts, what and whom markets are (and are not) designed to serve, and their uneven distribution.

## Spatio-market practices

In this section, we introduce the concept ‘spatio-market practices.’ We define spatio-market practices as those that contribute to the enactment of markets. In this respect, we follow Araujo et al. (2008, p. 8) and embrace a “broad sweep definition” that takes as its starting point the idea that markets have to be performed and that performances differ according to the worlds of market actors. By prefacing ‘market practice’ with ‘spatio,’ we intentionally foreground the idea that *all* market practices are spatial. This is the case because all market practices are here understood to depend on and be shaped by the mobilisation and enactment of various conceptualisations of space. These conceptualisations act to order everyday experiences and structure the wider gamut of social-material relations. This understanding takes inspiration from Lefebvre’s (1991, p. 416) argument that “nothing and no one can avoid *trial* by space.” Again, taking our lead from Lefebvre (1991), we suggest that the nature of such trials can be revealed by examining the performance of spatio-market practices, and related connections and dynamics.

Our conceptualisation of spatio-market practice reflects, more broadly, a weaving together of ideas generated in market studies and those linked with the ‘spatial turn’ in social theory (Merriman et al., 2012). A scientific and mathematical treatment of space, with roots in Euclidean geometry, long saw it conceptualised and treated as a dead void filled with objects (Foucault, 1980; Massey, 2005; Smith, 1984). Over the course of the twentieth century, however, scholars successfully brought space to 'life,’ conceptualising and showing how space does not exist ‘out there’ as an abstract entity, neutral background, or container defined by stasis, but is instead continually produced through social, political and economic activity and relations. As Beyes and Holt (2020, p. 5) write, “the spatial turn now figures prominently in human scientist circles, denoting a renaissance of ‘space’ as a conceptual and analytical category, and marking a renewed interest in the spatial nature of human experience.”

The spatial turn has seen the production of physical and representational space(s) and spatial concepts, such as site, place, territory, region, and scale, conceptualised and approached as outcomes of the performance and ordering of practices. Building on this positioning, our conceptualisation of spatio-market practices more specifically involves a synthesis of elements of Lefebvre’s (1991) and Harvey’s (1973, 2006) seminal works. Lefebvre’s (1991) influential work–*The Production of Space*–foregrounds the inescapably spatial dimensions of everyday practice, and for our purposes, market practices. Harvey’s (1973, 2006) work adds further analytical value, providing a set of dominant spatial categories (absolute, relative and relational), which are used day-to-day as part of the performance of practices and are thus understood here to be mobilised by market actors as part of market making.

Three core and related propositions underpin our conceptualisation of spatio-market practices. Firstly, market actors are always engaged in and responding to the production of, exchange in, and consumption of tangible and imagined market contexts. Secondly, these market contexts, be they corporeal or imagined, are direct emanations of the ordering and performance of market practices. Thirdly, the ordering and performance of market practices and related contexts of production, exchange and consumption, depend on the mobilisation of various conceptualisations of space. These conceptualisations are, furthermore, integral to and shape everyday experiences and the wider constitution of social, cultural, political and economic relations and socio-material formations. From this standpoint, challenges of understanding how markets are made, why, and whom for, are resolved by examining the ordering and performance of spatio-market practices, the contexts they produce and respond to, and how they do so. In the next section, we ground our propositions in reference to a set of key ideas and concepts that lie at the heart of Lefebvre’s (1991) and Harvey’s (1973, 2006) works.

### The spatial dimensions and dynamics of everyday spatio-market practices

**Diagram 1:**
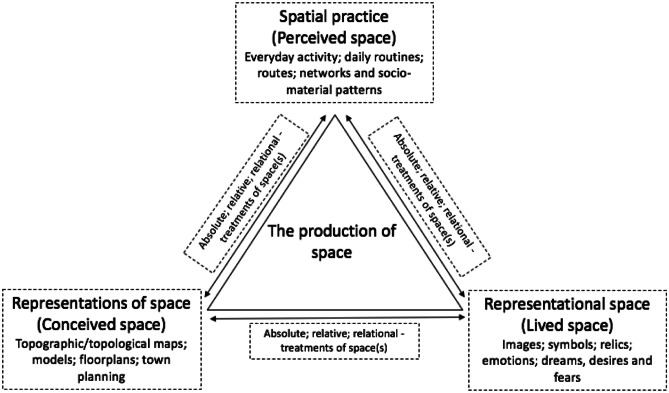
Lefebvre’s (1991) triad and Harvey’s (1973, 2006) three dominant treatments of space read in conjunction and overlayed

The three propositions noted align closely with Lefebvre’s (1991) and Harvey’s (1973, 2006) discussions concerning the spatial dimensions and dynamics of everyday life and social, cultural, political and economic relations. Lefebvre (1991) discusses these in reference to two overlayed conceptual triads (‘perceived-conceived-lived’ and ‘spatial practice-representations of space-representational spaces’). The ‘perceived-conceived-lived’ triad acknowledges and stresses the spatial dimensions of a social subject’s everyday experiences and actions. The overlaying of ‘spatial practice-representations of space-representational spaces’ emphasises that the ongoing production of space(s) involves a multiplicity of perceiving, conceiving and living social subjects that perform practices and together respond to and (re)produce physical and meaningful sites through organised and rhythmic patterns of activity (for a more detailed discussion of the rhythmic dynamics of everyday life see Lefebvre, 2004). Lefebvre (1991) explains that his conceptualisation of perceived space most closely aligns with spatial practice; conceived space with representations of space; and lived space with representational spaces. Harvey (1973, 2006) likewise centers ‘practice’ in his work, drawing specific connections between the enactment of practices, the mobilisation of three conceptualisations of space (absolute; relative; relational), and the uneven production of material and representational geographies. We discuss Harvey's (1973, 2006) and Lefebvre’s (1991) ideas in conjunction because they complement each other and specifically support attempts to decipher and reveal the everyday spatial dimensions and dynamics of market making through practice. Diagram 1 provides a representation of how we interpret and combine the authors’ ideas. In this section, we explain in more detail the links between the ideas captured in the diagram.

Emphasising the spatial dimensions of subjectivity, Lefebvre (1991) discusses the first element of his perceived-conceived-lived triad in reference to the social subject as a living, breathing, thinking and perceptive “body” (p. 40). As he explains, the existence of “social practices presuppose the use of the body: the use of the hands, members and sensory organs, and the gestures of work” (Lefebvre, 1991, p. 40). “Put in psychology’s terms, the realm of the perceived… [is] the practical basis of the perception of the outside world” (Lefebvre, 1991, p. 40). This perceptive and instinctive body, which encounters and helps make the outside world (e.g., buildings, sites of leisure, retail shops, roads, city neighbourhoods), is always caught up in and integral to the construction of social spaces through engagement in wider patterns of, what Lefebvre (1991, p. 38) terms, “spatial practice.” In this sense, the perceptive subjective body is embedded in and shaped by the social body it is a part of. Bodies do not, therefore, encounter or perceive objects abstractly but always through spatial practice and ‘in’ market contexts, be they linked with production, exchange and/or consumption.

By spatial practice, Lefebvre (1991, p. 33) specifically refers to the everyday collective routines of actors and the related production of “particular locations and spatial sets characteristic of each spatial formation.” It is, as Lefebvre (1991, p. 38) writes, “the spatial practice of a society that secretes that society’s space.” As means of example and in figurative terms, a supermarket is (like all marketplaces) a nexus of routine patterns of production, exchange and consumption, all of which involve multiple perceptive, active, mobile and productive bodies, engaged in the enactment of spatial practice and the related production of the site as a physical and meaningful formation. Key here is the idea that market practices do not happen *in* or *across* space in an abstract sense. Rather, market practices are always spatial because perceptive actors are inevitably involved in, shaped by, and take aim at the routine production of particular market spaces. The spatial practices that take aim at and help make a supermarket are, for example, different (at least in some identifiable ways) to those involved in the production of other market sites, such as homes, leisure venues, digital marketplaces, and those of worship, health, and education. The distinguishing physical and representational characteristic of such market spaces and the ability to identify them separately evidences this point.

Another linked dimension of the everyday spatiality of market practices lies in the conceptual capacities of social subjects and thus market actors, and how they handle, order, and make sense of the spaces they perceive and produce day-to-day. This analytic capacity in crucial to everyday action. As we go on to explain, it would not be possible, for example, to distinguish entities, situate them, track their movements, and describe where they lie in relation to each other, without conceptualising space in particular terms. It would not, moreover, be possible to produce socially and institutionally codified and instructive depictions of market spaces.

The conceptive capacities of social subjects are distilled in socially and institutionally codified and instructive depictions of space. Lefebvre (1991) calls such depictions ‘representations of space.’ Examples include, inter alia, topographic and topological maps, product portfolios, floorplans and planograms. These examples are always social artifacts because they are the product of shared values and logics deployed as part of producing space(s) to be disseminated, interpreted, engaged with and enacted.

The idea that perceptive bodies, embroiled in spatial practices, navigate and help to produce physical and meaningful spaces by drawing on various conceptions of space is complemented by Harvey’s (1973, 2006) discussion of ‘absolute,’ ‘relative’ and ‘relational’ treatments of space. Treated as absolute, “space… becomes ‘a thing in itself’ with an existence independent of matter” (Harvey, 1973, p. 13). As Harvey (2006, p. 272) notes, “absolute space is fixed, and we record or plan events within its frame.” Absolute space thus exists regardless of objects, as a void that can be filled with matter. Thought of in this sense, all manner of objects take up portions of absolute space. By means of example, the physical organisation of marketplaces, be they more traditional or digital, depend on viewing space as a void and something that can be filled with products and/or services. Amazon’s homepage, for instance, is managed and treated as an absolute space, within which certain tabs and products are distributed, ranked and presented. The physical organisation of a supermarket *– *the arrangement of tills, the ordering of aisles, the positioning of items*–*likewise depends on viewing space in absolute terms and arranging objects in the available ‘empty’ market space(s).

A key issue here is the constitution and the effects of absolute and contained market spaces. That is, how and what the conceptualisation, construction, and encountering of contained spaces do and do not permit. These possibilities and limitations logically differ across market practices and markets more broadly. In hypothetical terms, the absolute space that a website designer encounters and helps to produce enables and structures actions in different ways to those linked with a supermarket employee or those connected with managing international stocks and trades. In short, different market contexts, as necessarily produced, encountered and treated in ‘absolute’ terms, are not only the product of market activity but shape material and value-laden fields of action and possibility.

The second dominant conceptualisation of space is as relative. Based on this treatment, space exists “as a relationship *between* objects which exists only because objects exist and relate to each other” (Harvey, 1973, p. 13). The relative view of space acknowledges distance, its creation, and the idea that there are multiple geographies to everyday life and different forms of ‘near’ and ‘far.’ As Harvey (2006, p. 272) notes, “space is relative in the double sense: that there are multiple geometries from which to choose and that the spatial frame depends crucially upon what it is that is being relativized and by whom.” Without this view of space, it would not be possible to make topological maps, to measure relative distances, and plan and structure various forms of everyday market practices, which, crucially, involve decisions about placing market objects (e.g., products; people; distribution centres; stores) and measuring ‘the’ space between them.

To return again to the illustrative example of a supermarket, a relative view of space is crucial to the planning and careful design of the shop floor as an absolute space comprised of objects placed in qualified positions to each other. This is clear from the efforts that go into the positioning of different items and the related design of desired customer routes. The relative view of space is further critical for the everyday scheduling of deliveries and the picking and distribution of items for online orders. Key issues here include the production of specific topological market geographies, what and where market entities are relatively located and plotted, what it takes to navigate created and encountered distances, speeds of transaction, and who is (and is not) ‘on the map’ and granted access.

The third dominant treatment of space is as ‘relational.’ Space is conceptualised, in this instance, to be “contained *in* objects in the sense that an object can be said to exist only insofar as it contains and represents within itself relationships with other objects” (Harvey, 1973, p. 13). A relational view of space can be understood by turning once more to the supermarket example. An employee tasked, for example, with tidying up the store works with a relational view of space, picking up discarded items that do not belong on one aisle and taking them back to their ‘proper place.’ In this sense, items have meaning and are regarded as belonging to categorized aisles. Crucially, the task of tidying the shop floor would not be possible without viewing space as a meaningful and relational formation, which must be maintained, configured and produced in accordance with particular normative expectations and standards as distilled *in* objects. Such normative expectations and standards are captured in retail planograms, which depict where products should and should not be placed across shop floors, on shelves and in relation to each other.

As our anecdotal example suggests, markets are conceptualised and encountered as relational spaces when they are organised and experienced as socio-material arrangements of related entities, goods, and services in particular meaning-imbued contexts, which are shaped by and shaping of everyday market practices. Without a relational treatment of market spaces, it would not be possible to design or enact everyday market and marketing practices, which trade on multiple forms of distinction between socio-material entities. Key issues here concern the value-laden relational production, placement and perception of market entities and the related constitution and underpinning logic of various market contexts.

Returning to Lefebvre’s (1991) analytical triad, the everyday treatment of space in absolute, relative and relational terms is not only integral to how spaces are perceived but also to how they are ‘lived’ and produced more broadly as representational formations. As Lefebvre (1991, p. 39) writes, “space [is] directly lived through its associated images and symbols.” These images and symbols specifically overlay physical materials and arrangements thereof, giving meaning and forms of order that are interpreted, felt and reproduced by “inhabitants” and “users” (Lefebvre, 1991, p. 39). Without this overlaying, it would not (as we have explained in reference to a figurative supermarket) be possible to distinguish and arrange items to produce desired emotional affects. It would also not be possible to appropriately interpret, track and manipulate relative distances, movements and flows of items, services and customers across the absolute space of the shopfloor without a value-laden sensitivity of objects, relations and ‘spaces’ within ‘space.’

This lived and value-laden production of space is again a social process codified in what Lefebvre (1991) terms ‘representational spaces.’ These spaces “tend towards more or less coherent systems of non-verbal symbols and signs” (Lefebvre, 1991, p. 39), which are intelligible to specific cohorts and communities of practitioners. As Lefebvre (1991, p. 40) notes, “lived experience… may be both highly complex and quite particular, because ‘culture’ intervenes here, with its illusory immediacy.” This immediacy is manifest through the perceptive social body and practitioner that has been educated to conceptualise, decipher, experience, feel and also play a part in producing affective and representational spaces imbued with meanings and normative values.

Inspired by the works of Harvey (2006) and Schwanen (2019), Table 1,^1^ brings all of the ideas presented in this section together. As explained, Lefebvre (1991) captures the inescapably spatial dimensions and dynamics of subjective experience and social relations ‘at large’ by overlaying the perceived-conceived-lived with spatial practice-representations of space-representational spaces. We too overlay the ideas on top of each other in Table 1 below across the Y axis as part of acknowledging both the micro and macro, and subjective and shared, dimensions and dynamics of everyday spatio-market practices. We have also mapped Harvey’s (1973, 2006) discussion of three dominant conceptions of space across the X axis with Lefebvre’s (1991) triad in order to draw links between each author’s work and to help think about the different spatial dimensions and dynamics of everyday market practices. As noted, (and captured in Diagram 1), we see each element of Lefebvre’s (1991) triad and the relationships between them as mediated through the dominant treatments of space Harvey (1973, 2006) outlines. This is to say that the production of market spaces is never separate from practical treatments of space. Accordingly, perceived, conceived and lived components of spatial production and the wider societal ordering of spatial practices, representations of space and representational space, and the relationships between these features, are shot through with and shaped by absolute, relative and relational treatments of corporeal and imagined spaces. It is also important to note that the elements of Lefebvre’s (1991) triad and our overlaying of absolute, relative and relational treatments of space are co-dependent and entangled components–the perceived, conceived and lived are recursively connected; and absolute, relative and relational treatments of space are analytical categories that only have meaning in reference to each other. As we explain in the next section, it is important to work with each of the concepts introduced in tandem as part of examining spatio-market practices.

**Table 1:** Spatio-market practices—A diagram of cross-cutting ideas and example meanings

Spatio-market practices	Perceived (Spatial Practice)	Conceived (Representations of Space)	Lived (Representational Spaces)
Absolute	Encounters and experiences with objects in built market contexts (e.g., homes; workplaces; shopfloors; webpages; neighbourhoods; city quarters; cities; countries; territorial markets) Physical geographies; Boundaries and barriers; Closed markets; Open space	Market spaces conceptualised as voids and containers; Euclidean geometry; Topographic maps (e.g., captured in representations of fixed market geographies and segments); Metaphors of internment, openness and freedom; Plotting of ‘doing’ places and thus positionality; Descartes (‘*Cogito, ergo sum’ (I think, therefore I am)* ‘here,’ which is different from 'there')	Affective knowledge of one’s surroundings; Sense of attachment and security (or not) ‘in’ places of production, exchange and/or consumption; Fear of the ‘outside’ and of ‘others’ (or not), including competitors and market regulators; Sited sense of power or powerlessness in ‘a’ market context
Relative	Flows and circulation of objects, people, information, knowledge, services, trade and capital; Perceptive and alternating speeds	Markets spaces conceptualised as made up of points; Topological maps (metro system maps; supply chain maps; flow charts; Gantt charts; route plans); Distance between points; Metaphors of movement, motion and mobility; Time–space compression (‘annihilation of space by time’)	Unease over travel (e.g., fear of not being on time—being late for an important meeting or event; not delivering on time; frustration due to congestion, bottlenecks and queues that hamper production, exchange or consumption); Excitement of moving into new spaces of work and/or play; Anxiety over increased competition; Exhilaration/fear over increased speeds of production and/or exchange
Relational	Relationships between human and non-human actors ‘in’ market spaces; Sensations (sight, sound, taste and smell) stimulated through spatial encounters; Value perceived as that emergent of associations between objects (e.g., land values; products on a shelf)	Market spaces conceptualised as made up of relationships contingent on meaningful objects (e.g., planograms; product portfolios; consumer journey maps); Market and product narratives distilled as an outcome of social-material dependent on the relational meaning of market entities; Future hopes and fears dependent on such relationships; Strategies for change made possible and highly conditional	Value-laden wants, needs and desires embedded in and fulfilled through spatial encounters with marketized objects; Aims and objectives definable in reference to each other and other socio-material market entities and relationships; Memories and dreams made given life; Meaning-imbued marketized sites of discipline, happiness, love, worship and mourning encountered, learnt and (re)produced through ongoing and meaning-imbued practice

By bringing the ideas together as we do in Table 1, we have specifically attempted to think through how the perceived, conceived and lived dimensions of everyday experience and wider social relations can be fruitfully disaggregated, interpreted and explained in reference to the actions of market actors and how they treat space along absolute, relative and relational lines. The main reason for doing this is to support empirical enquiry, and thus to help researchers explicate the spatial dimensions and dynamics of market practices. We consider the theoretical and empirical implications of working with the ideas introduced in more detail in the coming section.

### Theoretical and empirical implications

Our conceptualisation of spatio-market practices has several implications for future empirical enquiry. The first is that markets should be treated as products of spatio-market practices. Studying markets is, from this perspective, about examining spatio-market practices and how spaces are perceived, conceived, and lived through practice, be they, in Kjellberg and Helgesson’s (2007) terms, 'normalizing,' 'representational,' or 'exchange-oriented.' The second implication is that there is a multiplicity of spatio-market practices and market spaces. This is both a product of the multiplicity of market practices and the mobilisation and effects of different treatments of space by market actors. Together, these implications link with a third, which is that empirical research is necessary to construe the spatial dimensions of particular market practices and what these practices reveal about the logic of specific markets. In this regard, Harvey’s (1973, p. 13) following suggestion holds particular resonance:

*The problem of the proper conceptualization of space is resolved through human practices with respect to it. In other words, there are no philosophical answers to philosophical questions that arise over the nature of space–the answers lie in human practice. The question “what is space?” is therefore replaced by the question “how is it that different human practices create and make use of distinctive conceptualizations of space?”*

This means that absolute, relative, and relational conceptualisations of space have to be revealed in action. As Harvey (1973, p. 13) explains “space is neither absolute, relative or relational *in itself,* but can become one or all simultaneously depending on the circumstances.” Revealing circumstances is an empirical endeavour and it is only through research that it becomes possible to reveal how, when and why particular treatments of space are mobilised and to what effect. Likewise, the mobilisation of spatial concepts, such as ‘place,’ ‘territory,’ ‘region,’ ‘scale,’ ‘local’ and ‘global,’ and the related use of absolute, relative, and relational treatments of space, should be approached as products of the performance of various market practices that have to be distinguished, in design and effect, through research. (See, for example, Adams, 1996; Brenner, 1997; Cox, 1995; Marston, 2000; Moore, 2008 who reveal and explore the effects of the social production of spatial scales through practice).

Key questions concern: which treatments of space are mobilised as part of the performance of market practices, how so, and with what consequences? How do specific treatments of space underpin and shape the perceptive capacities of market actors, the wider ordering of spatial practices, and the physical construction of social sites? And how do different treatments of space link with the related production and experience of ‘lived’ and meaning-imbued market spaces and the broader configuration of representational spaces?

These types of questions admittedly need to be refined in reference to particular empirical topics. They are, nevertheless, the types of questions that, if addressed, promise to shed new light on what it takes to make markets function, and what and whom such formations serve. To maximise the value of insights generated, it is important to avoid the pitfall of treating Lefebvre’s (1991, p. 40) triad “as an abstract model’’ and of neglecting the simultaneous or intermittent influence of absolute, relative and relational treatments of space in action. To do this, it is crucial to remain sensitive to the perceived, conceived and lived dimensions of market practice and the broader socio-spatial dynamics of market making. Failing to do so runs the risk of missing crucial details regarding the spatial dimensions and dynamics of market making and associated effects.

A range of methods could be drawn on to address the types of questions outlined and we purposefully shy away from any prescriptive suggestions. Having emphasised the heterogeneity of spatio-market practices, both in terms of the types of practices performed, the concepts drawn on, and the related types of spaces perceived, conceived and lived, it would be amiss to set out a prescriptive set of methods. Rather, we call for engagement with a range of methods that help to reveal the spaces of market practices, how these are made, and related social, economic, and political implications. In this regard, absolute, relative, and relational categories of space provide a useful point of departure. We draw on these categories in the coming section, within which we provide an illustrative case that brings a collection of secondary sources together as part of an alternative and spatially-oriented account of the growth and legitimacy of the American casino gambling market. We provide this short illustrative account in order to ground the concept and to make cursory inroads into demonstrating its value for market-oriented research.

## The American casino market: spatio-market practices, market growth and constituting legitimacy

The number of casinos in America grew exponentially over the past century (Humphreys, 2010). Casino gambling moved, during this time, from a one-state marginal business to a twenty-nine state mega-industry (Garrett, 2003; Humphreys, 2010; Moehring & Green, 2005). In Nevada, a state that legalised gambling in 1931, there were two hundred and ten casinos by 2001 (Garrett, 2003). These generated USD 9.5 billion (Garrett, 2003). “75,000 electronic gambling devices (EGDs)… and 3,300 table games [took] up 3.3 million square feet of casino floor space” across the state by 2001. As of 2003, the casinos in Nevada annually served an estimated thirty-five million tourists who visited and utilised the hundred thousand plus hotel rooms (Garrett, 2003). In Atlantic City, where gambling was legalised in 1976, nearly USD 4.3 billion was generated annually by 2003, thanks to the arrival of thirty-two million visitors. These frequented the twelve thousand hotel rooms, using the thirty-seven thousand electronic gambling devices and more than twelve hundred table games, which took up an estimated 1.3 million square feet of casino floor space (Garrett, 2003). The market’s growth has not only involved the development of land. Beginning first in 1991, Riverboat casino gambling quickly spread throughout America’s Midwest. By 2003, there were riverboat casinos in Indiana, Mississippi and Missouri (Garrett, 2003). Riverboat casinos are sizable, with many having “more than 1,500 hotel rooms” (Garrett, 2003, p. 4). The American casino market has thus grown rapidly, both in terms of its economic size, footprint, and the number of actors (human and non) involved.

In the below sections, we will show how this growth has been a spatial affair, played out through the enactment of various spatio-markets practices. Our account takes inspiration from Humphreys’ (2010) influential paper. Yet, we specifically draw attention to the socio-spatial dynamics and dimensions of market making. Humphreys’ (2010) excellent account of the growth of American casino gambling hinges on an examination of the market’s discursive framing in national newspapers and industry publications since 1980. The author explains the market’s growth in reference to processes of legitimisation, constituted through the framing work of key stakeholders (e.g., regulators, public policy activists, and financial investors). As she writes, these actors navigate and inform the social and political “environment that exists outside the… industry,” creating a legitimised market “space” (Humphreys, 2010, p. 1). This market ‘space’ remains purely discursive in Humphreys’ (2010) work.

From a Lefebvrean perspective, the analysis concerns the constitution of “representations of space” (Lefebvre, 1991, p. 39). And, based on the Kjellberg and Helgesson’s (2007) categorisation of market practices, Humphreys (2010) focuses on those connected with representational work. The spatial dimensions of the market’s growth are reduced, in turn, to contestations over the market’s meaning across discursive spaces. Discussion of a market “environment that exists *outside* the… industry” (Humphreys, 2010, p. 1) (emphasis added), also means that the market’s spatiality is presented in exogenous terms and as something ‘out there.’ These features of the article leave room to discern the ‘more-than-discursive’ spatial constitution of the market over time. An opportunity also remains to fold the “outside” inside the market practices of key actors and in so doing reveal what it took to make the market legitimate, who was involved, and further explore what these details suggest about the practical, social, political and economic dimensions of the market’s growth.

In a cursory sense and based on details discussed concerning the market’s growth, over time, decisions had to be taken about the location of casinos, the design and scale of buildings, carparks and other service infrastructures, the layout of casino floors, and, inter alia, the types of games available. These decisions and others alike involved the production of discursive and physical spaces and were part of attracting customers, accruing capital and shaping the market’s growth and constituting legitimacy over time. Building on these suggestions and the details outlined, it is appropriate to interpret the market’s growth and legitimacy as spatial affairs. To dig deeper into and discern the spatial dimensions of the market’s growth and legitimacy, it is useful, however, to explore the perceived, conceived, and lived spaces of market actors and, in a related fashion, how conceptualisations of space(s) are integral to the market’s constitution. We now turn to a selection of written and visual sources that help elicit such details in reference to particular market practices.

### The spatial dimensions of investment practices and legacies of legitimacy

**Fig. 1:**
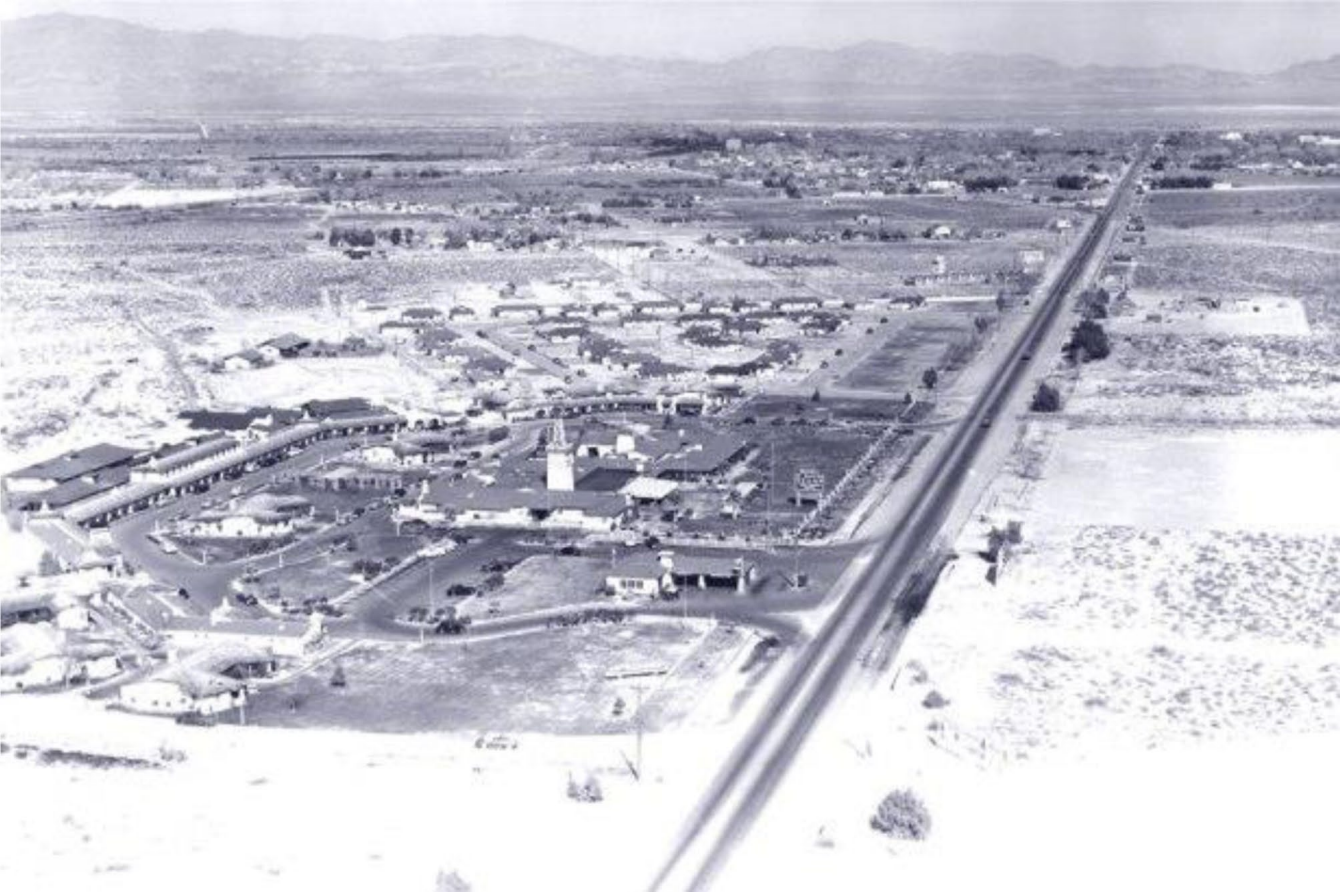
An overview of the El Rancho property before the competitors popped up (https://jhgraham.com/2018/02/24/el-rancho-vegas/)

**Fig. 2:**
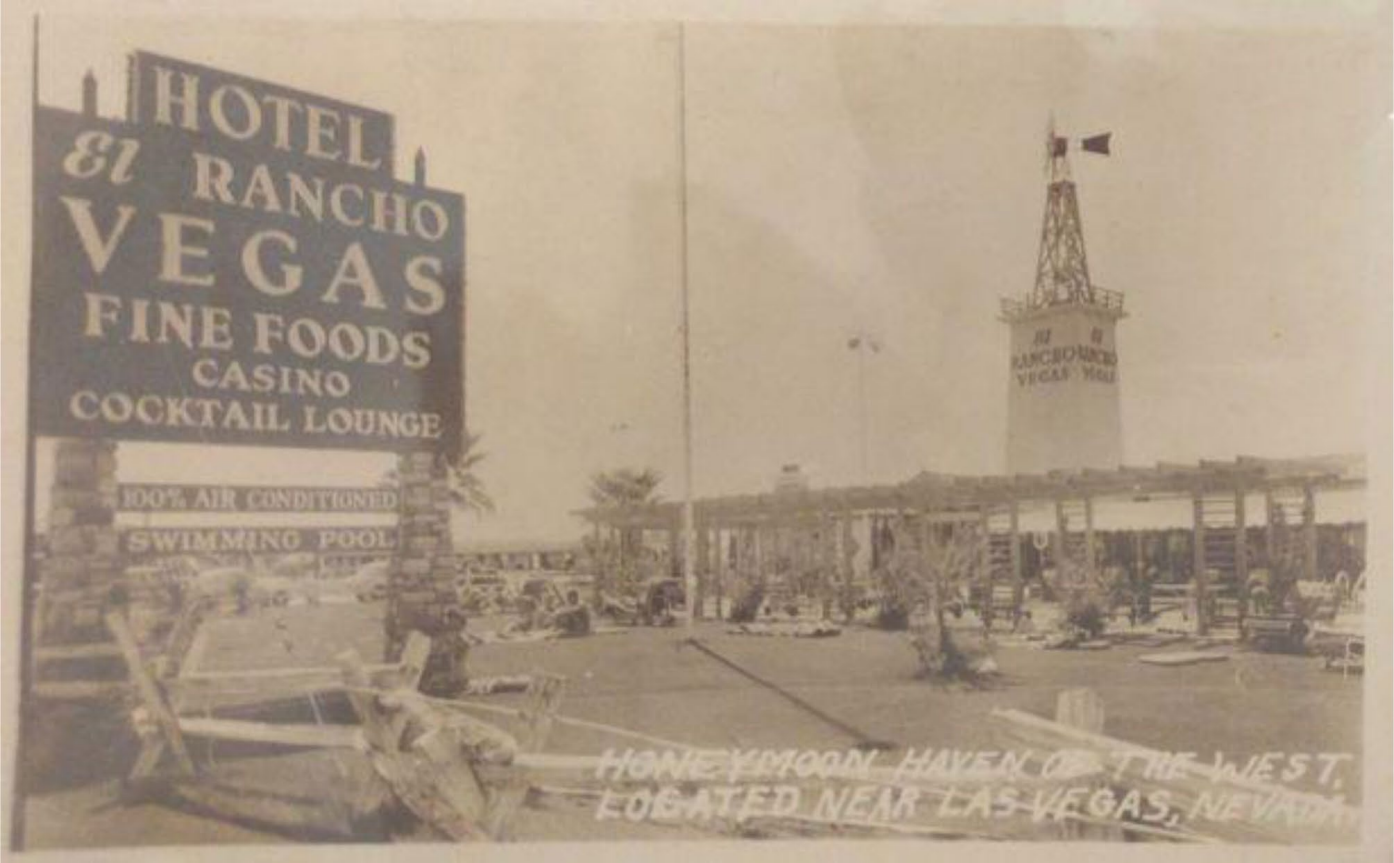
Postcard view of El Rancho Vegas, with sunbathers on the lawn between the pool and Highway 91 (https://jhgraham.com/2018/02/24/el-rancho-vegas/)

Upon legalisation, the view of Nevada was transformed for investors and developers*–*a new field of opportunity opened up. To reap the benefits, hoteliers, such as Thomas Hull, who built El Rancho, the first casino-hotel in Las Vegas, mobilised an absolute view of space (Figs. 1, 2). Conceiving and perceiving the state as a container, filled with plots of land, Hull was able to make an informed decision about where to develop his extravagant project.

Though encouraged by city officials to build downtown, Hull decided to buy a less expensive and larger plot of land in the desert, in the neighbouring Clark County (Moehring & Green, 2005). This decision was shaped by his lived experience as a hotelier and owner of multiple El Rancho resorts spread out across the country. Thanks to this history, he was looking (in absolute terms) for “space” (Moehring & Green, 2005, p. 109). This was “the real advantage of moving to the outer suburbs” (Moehring & Green, 2005, p. 109). “On his desert acreage [,] Hull had room to build a spacious casino, coffee shop, buffet, gourmet restaurant, pool, lush lawns and gardens, and, most important, parking for four hundred cars” (Moehring & Green, 2005, p. 109). Conceiving and perceiving of space in absolute terms, Hull was thus able to view the state as a field of opportunity. This helped him to take steps toward making an informed investment decision about the plot of land, which was nested in a wider field of possibility. This decision was a product of his informed spatio-market experience and imagination.

**Fig. 3:**
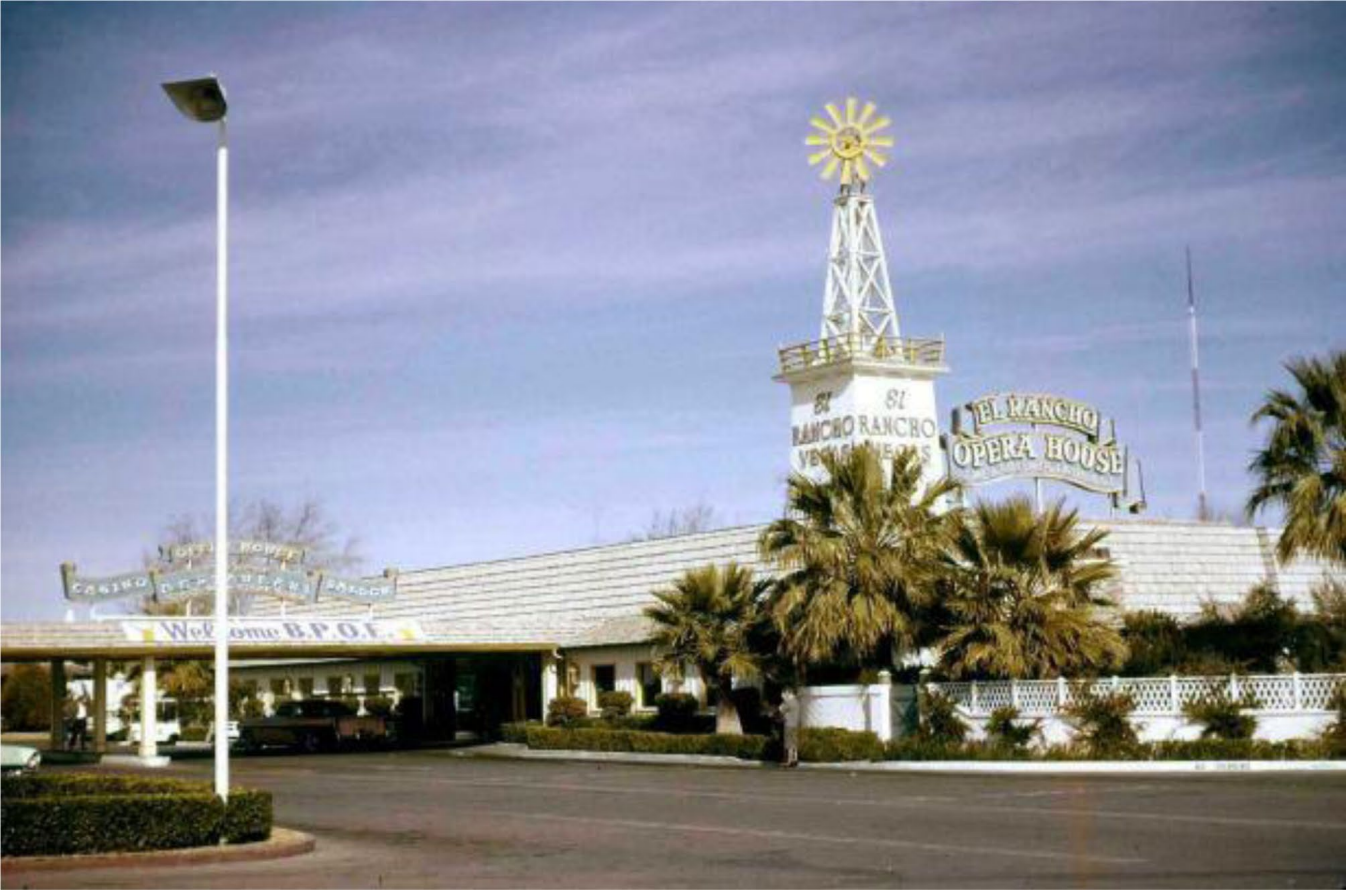
The carport entrance (added in 1959) (https://jhgraham.com/2018/02/24/el-rancho-vegas/)

Hull’s investment decision also involved thinking about the relative placement of the plot of land in reference to Las Vegas and key infrastructure networks. He needed ‘space,’ but in relative terms, he also needed customers and was careful to acquire a plot of land on Highway 91, which connects Las Vegas and Los Angeles. This meant he was not only free from the typically narrow confines of city lots but was close enough to Las Vegas and served by a connecting road network that enabled customers to be drawn into the casino-hotel resort regardless of whether they were traveling by car, bus, train or air (Fig. 3).

The relative view of space mobilised by Hull was also shot through with relational and meaning-imbued judgments. “As a southern Californian, Hull understood that the growing dominance of cars, trucks, and buses made the highway more important than the railroad station for delivering supplies and guests” (Moehring & Green, 2005, p. 109). Viewing space in relational terms, he visualised the desert plot as a point in space that could not be “understood by appeal to what exists only at that point” (Harvey, 2006, p. 274). The viability and value of the plot also depended “upon everything else going on around it” (Harvey, 2006, p. 274). This included not only the city as a hive and nucleus of activity but also dominant modes of transport and the growing access to and ownership of motorised vehicles. Such technologies, in a very relational sense, supported a judgment that was based on viewing the El Rancho development in relation to a set of figurative and connected meaningful ‘objects,’ including the city, road infrastructure, and forms of transportation. These objects, which only acquire meaning in relation to each other, informed and permeated the project’s viability and value.

**Fig. 4:**
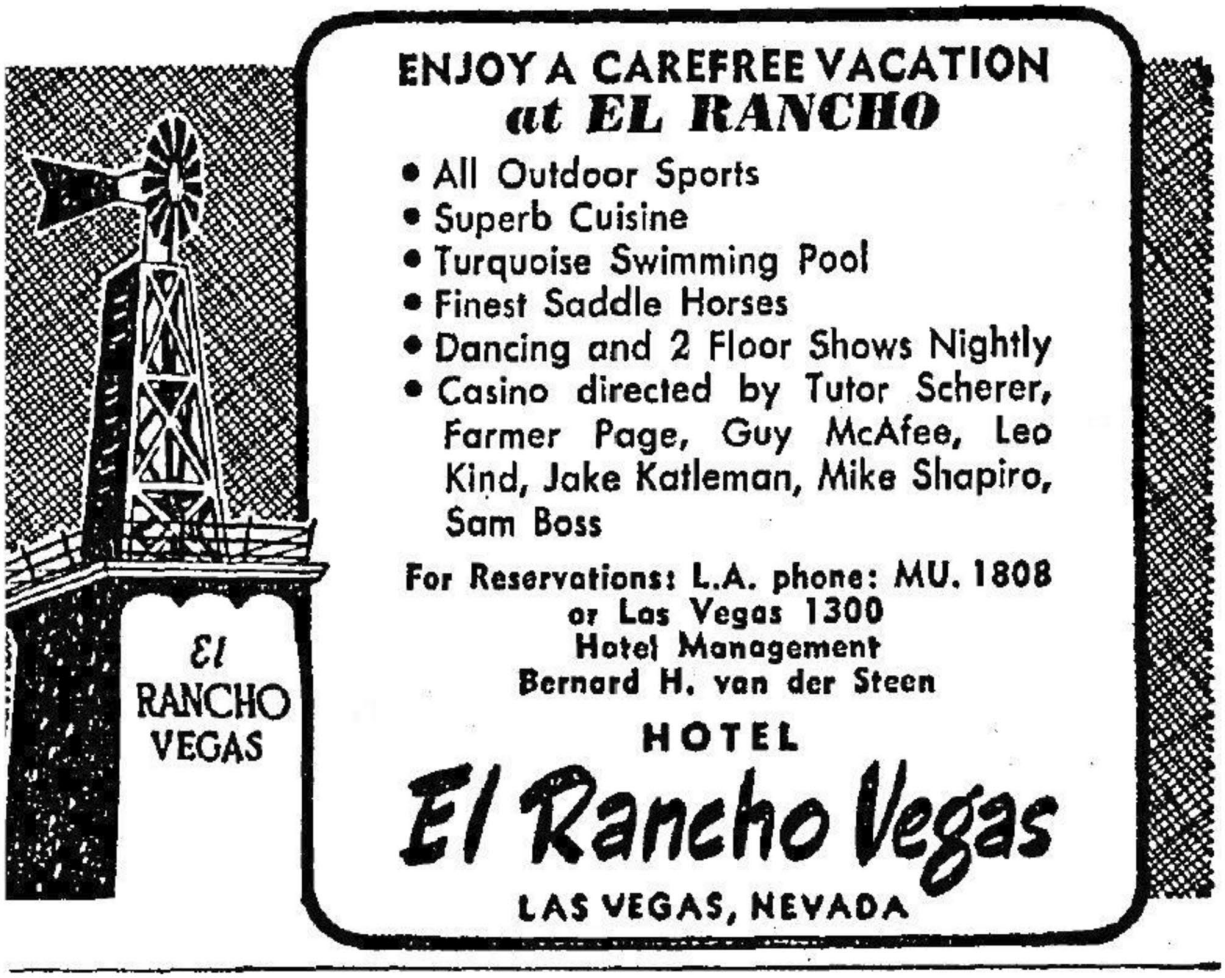
El Rancho casino-hotel resort and spa amenities (https://jhgraham.com/2018/02/24/el-rancho-vegas/)

From this example, we can see how the first casino-hotel resort development in Las Vegas involved the mobilisation of different conceptualisations of space, enacted through Hull’s spatio-market practice and associated perceptive, conceptive and lived experiences. Without deploying a set of spatially sensitive analytical tools, the El Rancho project would not have been possible. This spatial work is all the more significant as the El Rancho stood in stark contrast to the small casinos, card rooms, and bingo parlours that first took advantage of the market’s legalisation in Nevada (Moehring & Green, 2005). The El Rancho instead weaved together opportunities to gamble, drink, eat, enjoy entertainment shows, relax by the pool, and partake in excursions. It thus formed a nexus of spatial practices that helped constitute public acceptance (Fig. 4). Moreover, the new resort-oriented standard carried with it heavy meaning-imbued and normative expectations that shaped future investment decisions (Moehring & Green, 2005) and the production of the market as a broader discursive, legitimate, and spatio-material entity.

Beyond the ‘market,’ Las Vegas’ urban form and meaning can also be read through this spatially sensitive lens. Hull’s El Rancho specifically acted as an archetype for regeneration across the city as an economic hub, with the new model imprinted in and dependent on the production of a circuitry of roads, casinos, restaurants, bars, and other entertainment locales, anchored largely in resort developments (Moehring & Green, 2005). The market’s wider growth and legitimacy cannot be detached from this history and the spatio-market practices of actors like Hull, who helped constitute the more socially palatable casino resort as a grounded and discursive formation.

### The spatial dimensions of casino design and staying legitimate

**Fig. 5:**
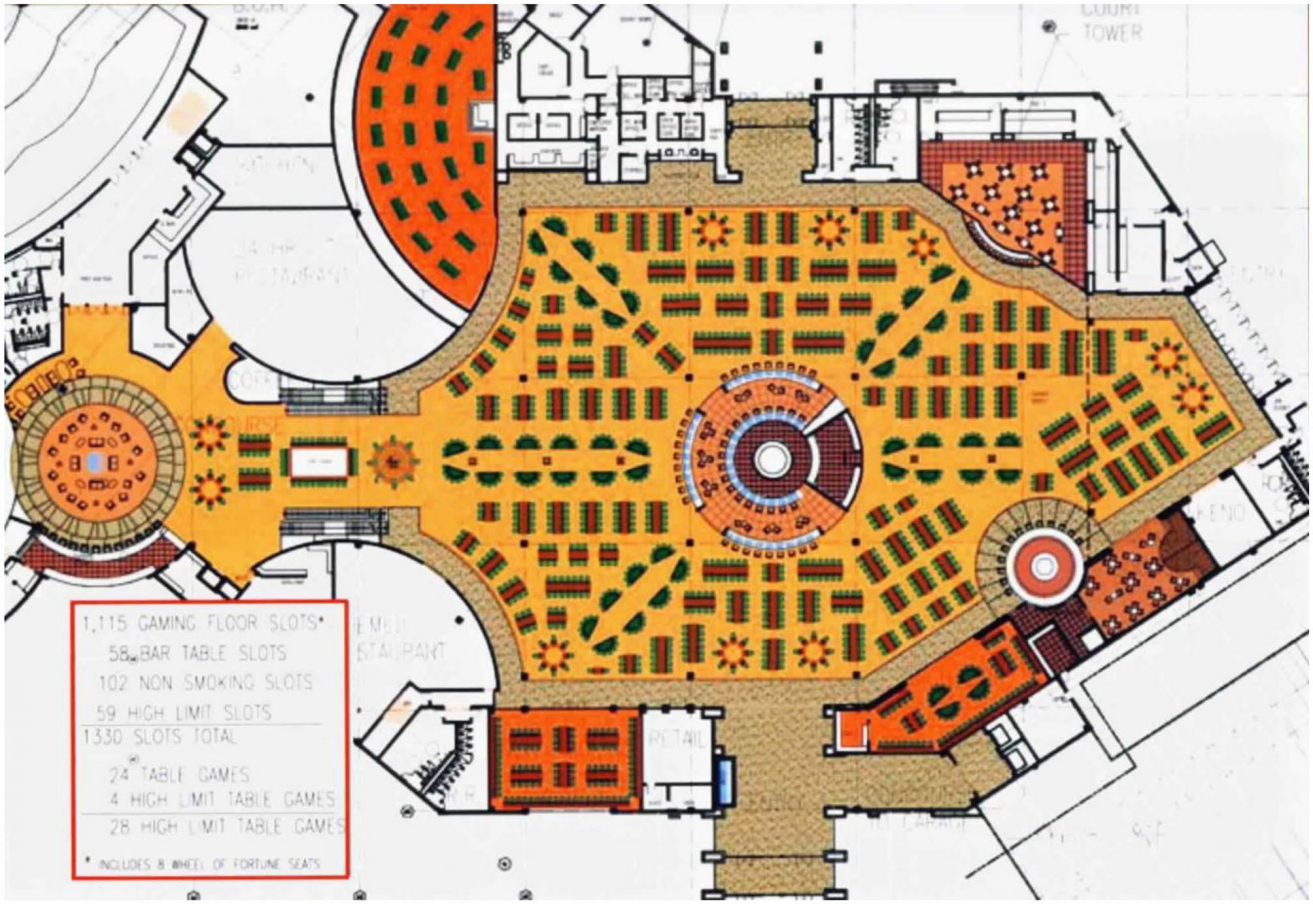
Wild Horse Pass Resort and Casino in Phoenix (http://www.nextindesign.com/master_planning?epik=dj0yJnU9Q2NhLUwtNWl5SE10WnNvSFhGWmlGSVJ5TU5QdTVqaUUmcD0wJm49dk5rdThKa1J0UElpNkl4RWxKbzRBZyZ0PUFBQUFBR0FLNHk0)

The spatial dimensions of the American casino market’s growth and legitimacy can also be traced through the spatio-market practices of actors involved in designing the interior of casino-hotel resorts. The work of Bill Friedman (2000) is particularly revealing. Friedman was a gambler who turned his attention to distinguishing a set of key casino design principles aimed at keeping gamblers gambling as long as possible. Over a twenty-year period, he visited approximately eighty casinos across Nevada. The result was a design consultancy business and a six-hundred-and-thirty-page book in which thirteen design principles are laid out. These became a go-to for casino managers and architects. The principles are as follows and the example floorplan of the 2009 Wild Horse Pass Resort and Casino in Phoenix (Fig. 5) sees them distilled in design:

- Principle 1 – A physically segmented casino beats a completely open barn
- Principle 2 – Gambling equipment immediately inside casino entrances beats vacant raised entrance landings and empty lobbies
- Principle 3 – Short lines of sight beat extensive visible depth
- Principle 4 – The maze layout beats long, wide, straight passageways and aisles
- Principle 5 – A compact and congested gambling equipment layout beats a vacant and spacious floor layout
- Principle 6 – An organized gambling equipment layout with focal points of interest beats a floor layout that lacks a sense of organization
- Principle 7 – Segregated sit-down facilities beat contiguous ones
- Principle 8 – Low ceilings beat high ceilings
- Principle 9 – Gambling equipment as the décor beats impressive and memorable decorations
- Principle 10 – Standard décor beats interior casino themes
- Principle 11 – Pathways emphasizing the gambling equipment beat the yellow brick road
- Principle 12 – Perception beats reality
- Principle 13 – Multiple interior settings and gambling ambiances beat a single atmosphere throughout

Each of the principles involves first treating a casino as a somewhat fixed and absolute entity, within which objects and opportunities to gamble can be distributed. The placement of such objects and opportunities involves making careful judgements about their relative positioning. These decisions, which depend on conceptualising the casino market in relative terms, are crucial to producing the ‘right’ focal points, pathways, flows and speeds of production, exchange and consumption. The principles also reflect a deeply relational treatment of space, with the qualified placement of objects and gambling opportunities recognising that casino floors are greater than the sum of their parts, being lived and experienced as meaningful sites of consumption. This sum depends on making calculations about how objects combine to produce desired emotional affects and behaviours. Indeed, an appreciation for the sensitivity of humans as relational actors lies at the core of Friedman’s (2000) work and more broadly across what is termed in the industry ‘casino design psychology’ (Schüll, 2014). Friedman’s (2000) principles and other contributions to the field of casino design psychology specifically reflect an understanding that humans are living and spatially perceptive actors, with identities, emotions, expectations and desires that are responsive to, shaped by, and constitutive of socio-material spaces and sites of exchange. The market’s handling and organisation and its public acceptance reflects and depends on appreciating and feeding such spatial sensitivities.

**Fig. 6:**
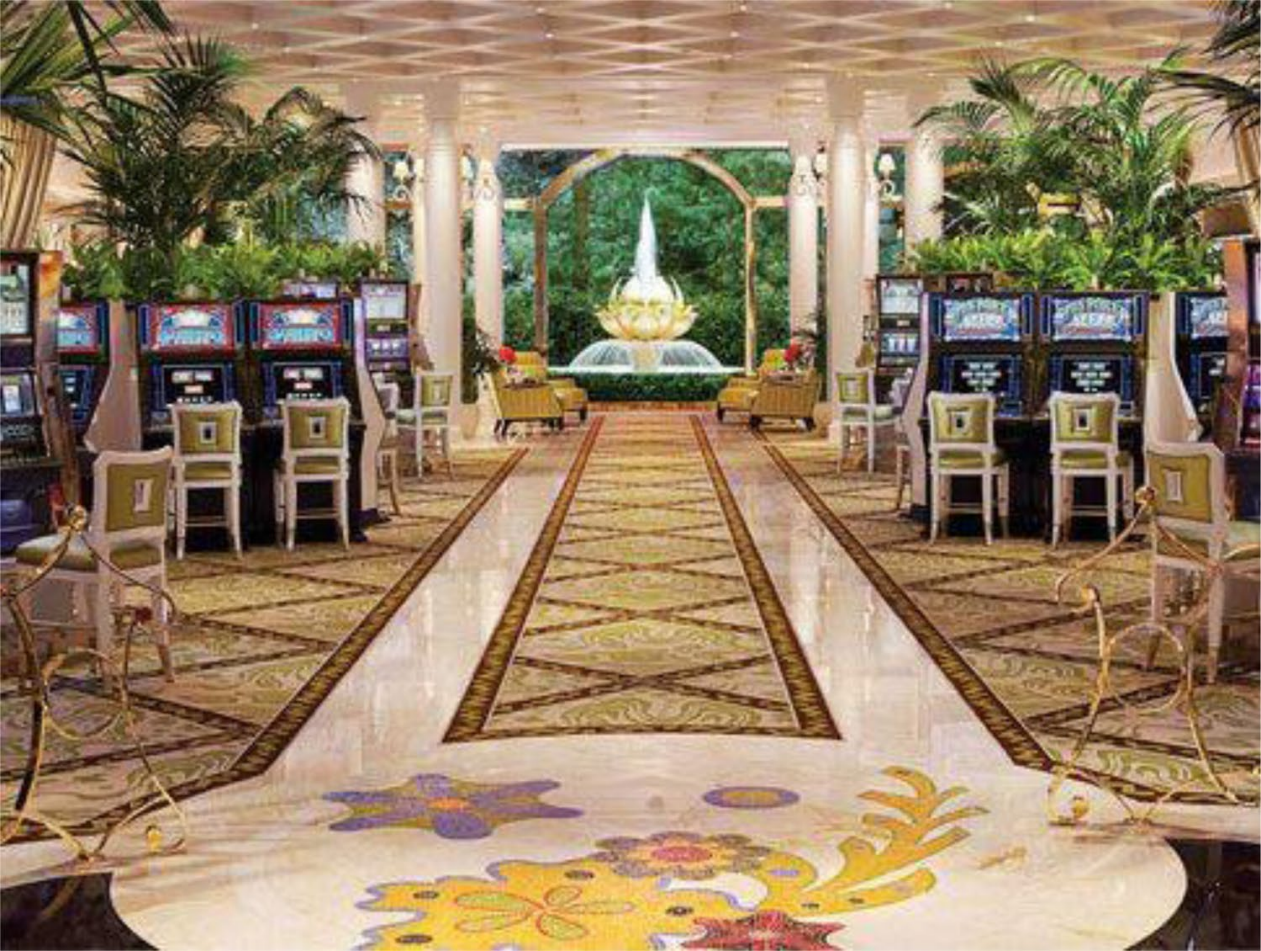
The redesigned high stakes slot machine room at the Wynn Las Vegas mega resort (https://vegasmagazine.com/channels/personalities/insights/interior-sketches)

Sensitivity for the relational dynamics of ‘lived’ spaces is particularly clear in unsuccessful and successful attempts to redesign casino floors. Famed casino designer Roger Thomas–the man who has led the redesign in Las Vegas along radically different lines to Friedman’s (2000) principles–speaks of the unsuccessful design of a high stakes slot machine room at the Wynn Las Vegas mega resort (Lehrer, 2012). Monitoring falling returns, he realised that the ‘clubby’ room, synonymous with “bourbon, testosterone, and cigars” (Lehrer, 2012), was very ‘male.’ This was an acute problem because men were not playing the high stakes slot machines. In response, he designed a “place where a lady might feel comfortable” (Lehrer, 2012). Mirrors, floral arrangements, Italian marble flooring, sculptures, water features, and a new colour scheme were introduced to achieve the desired effect (Fig. 6) (Lehrer, 2012). Thomas thus brought together an ensemble of materials, lights, sounds and scents, in an absolute space, relatively and relationally arranged, in order to create an experience, premised on a figurative and objectified image of a ‘female’ customer.

Such examples reveal the careful and thoughtful spatio-market practices and spatial sensitivities of designers involved in and integral to the ongoing production of the casino market as a stable and legitimate formation. These practices and sensitivities are recursively distributed across multiple actors and their connected capacities and roles. They also change over time, echoing changing trends, flows, arrangements and value systems. The market’s legitimacy, in turn, has various histories. We traced one line of enquiry above through to the spatio-market practices linked with the decision to invest in the eventually paradigmatic El Rancho resort. Crucially, legitimacy also has to be cultivated continually and this unfolds through the ongoing performance of various and intersecting spatio-market practices, such as those (but not only those) aimed at interior design. Accordingly, the market’s growth and legitimacy are not made in ‘a’ place, but rather through the everyday and simultaneous production of multiple connected material and discursive places, configured through and in accord with the dynamics of market practices and their related spatial dimensions. Table 2 captures and extends several insights presented in reference to Harvey's (1973, 2006) and Lefebvre's (1991) key conceptual ideas.

**Table 2:** Spatio-market practices that made the American casino market

Spatio-market practices and the American casino market	Perceived (Spatial Practice)	Conceived (Representations of Space)	Lived (Representational Spaces)
Absolute	Investors encountering Las Vegas, the layout of buildings, connecting service infrastructures, where opportunities lie to develop land; Casino managers meeting customers and handlining technologies across bounded sites of exchange that have to be maintained; Customers confronting, inter alia, casino floors, hotel rooms, concert halls, restaurants technologies and other people	Hoteliers, including Thomas Hull, look for ‘space’ by treating Nevada as a container, made up of plots of land, which offer opportunities to invest and accrue capital (e.g., captured in land use planning maps and other topographic maps); Casino design theorists like Bill Friedman and designers treating casinos as bounded containers within which various market objects, technologies and opportunities can and need to be built and distributed (e.g., distilled in architectural design plans)	Investors’ feelings of security (or not) in reference to their investment in a plot of land; Managers’ fear (or not) concerning ‘outside’ competitors or changing regulations; Casino designers’ experiences of rooms and what they elicit; Customers’ sense of comfort (or not) *in* a casino or specific casino room (e.g., female experiences of discomfort in a ‘clubby’ and overtly masculine room)
Relative	Investors, managers and staff encountering flows of customers, goods and services across the casino floor, hotel and along wider supply chains that reach out beyond ‘the’ site; Customers seeing and being caught up in flows of people, goods, money and other objects moving across casino floors, around, in and out of hotel rooms and ‘the’ city	Careful investment decisions about the relative position of and distance between casino hotels in reference to major roads, train stations, airports, other service infrastructures (e.g., water, electricity), distribution centres, competitors and hubs of entertainment Project Gantt charts and topological maps, such as schematic diagrams linked with service networks; The purposeful placement of market objects (gambling tables, slot machines, chip exchange stations, bars, toilets, the ‘pit,’ hotels rooms, concert halls, staff, customers) relative to each other to produce the ‘right’ focal points, flows and speeds of production, exchange and consumption (e.g., distilled in fine grain casino floorplans and customer route planning guides)	Investor’s unease over distance and travel (i.e., how will suppliers, staff and customers get to a casino); Casino designer and managers’ frustrations over possible and real bottlenecks in supply and queues fettering exchange; Customers lived experiences getting to and navigating casino hotels, moving from place-to-place, and between sites of consumption
Relational	Practitioners’ sensations (sight, sound, taste and smell) stimulated through spatial encounters on the way to and in a casino resort (e.g., Designer and customer perceptions of a ‘too clubby’ male-oriented gambling room premised on atmospherics and design aesthetics; investor perceptions of seedy and disreputable sites)	Investors seeing plots of land as opportunities configured in reference to other market ‘objects’ (e.g., El Rancho’s viability was, as shown, measured in reference to the ‘car’) distilled in project planning documents and in, for example, business plans and applications for loans; Designers treating the casino floor as made up of meaningful and related objects (e.g., tables, slot machines, lighting and heating technologies) and associations which, in combination, help to produce the ‘right’ or ‘wrong’ emotions, desires and consumer behaviours (i.e., the ‘clubby’ room was a place that needed to be re-designed and populated by materials, lights, sounds and scents to make it more feminine). These are seen, for example, in ‘themed’ representations of casino floorplans and fine grain interior design proposals	Investors’ value-laden judgments about the ‘natural’ beauty of a location and the representative qualities of the casino’s design and the clientele it brings in; Designers’ sensitivities to the meaning and effects of market objects in particular contexts and what these, in combination, produce (e.g., aesthetic feelings associated with lighting, sound and scent in context); Customers’ experiences, sensitivities and emotional expectations (e.g., feelings of love, elation, thrill, joy and agony) as felt in relation to encountered or dreamt up market objects (e.g., symbolic ornaments, flowers, lights, carpets, slot machines, gambling tables) and contexts (e.g., casino ‘pits,’ themed hotel rooms, restaurants, bars, nightclubs and concert halls)

## Conclusion

We have contributed a means of conceptualising the always and everyday spatial dimensions of market practices and market making. As others have argued, space is typically treated as a neutral backdrop or abstract environment in market-oriented research (Alvarez León et al., 2018; Castilhos & Dolbec, 2018; Castilhos et al., 2017). This being the case, the idea that markets are spatial formations is neither novel nor controversial. Pre-existing spatial concepts*–*site, place, territory, region, scale, network*–*have increasingly been used across marketing theory and economic geography to explain the broader organisation and politics of markets (see, for example Berndt & Boeckler, 2011; Castilhos et al., 2017; Alvarez León et al., 2018; Ashton & Christophers, 2018; Hall, 2018; Castilhos & Dolbec, 2018; Vicdan & Hong, 2018; Lloveras et al., 2018; Roux et al., 2018). In market studies, where everyday performativity is emphasised through a focus on practice, the spatiality of markets is implicitly suggested through discussion of ‘context’ and ‘distribution’ (Araujo, 2007; Finch & Geiger, 2010; Kjellberg & Helgesson, 2006; Stigzelius et al., 2018). We build on and add subtlety to these contributions by introducing the concept ‘spatio-market practice.’ This concept sees the spatial dimensions of market making folded inside the practices of market actors. This move accepts that practitioners cannot avoid “trial by space” (Lefebvre, 1991, p. 416). It also recognises that market actors necessarily draw on various spatial concepts as part of performing and ordering their work and markets more broadly.

The value of the concept lies in its empirical application. Through a short illustrative case, we articulated an alternative account of the American casino market’s growth and legitimacy. We showed that the market’s growth and wider public acceptance were thoroughly and inescapably spatial affairs. In other words, the market was created and enabled through practices and conceptualisations executed in reference to various enactments of space(s) (i.e., sited developments and the careful design of casinos to make the market accessible and acceptable). More specifically, we revealed how decisions about where and how to make a market, and whom and what to serve, are anchored in the spatio-market practices of market actors and how they perceive, conceptualise and live market spaces. In this regard, absolute, relative and relational treatments of space, as configured through the performance of spatio-market practices, permeate the uneven constitution and functioning of sites of production, exchange and consumption. Indeed, the enactment of such conceptions is integral to the everyday performance and framing of markets as physical and discursive formations. The emergence of casino resorts and the making of more ‘feminine’ gambling rooms evidence this suggestion.

By introducing the notion of spatio-market practice, we hope to stimulate further inquiry into the spatial dynamics of market making. Fruitful avenues for research may include, for instance, those linked to contemporary forms of digital technologies and devices. The sense of boundless movement and cloud-based services and exchanges granted by these technologies raise interesting questions about the making of ‘digital’ spaces, what this takes and the relationships, purposes and communities they do and do not serve. Another contemporary area of study relates to the Covid-19 pandemic. This quite unprecedented period of disruption has seen a mass reordering of socio-spatial relations, with social distancing becoming a norm, various boundaries hardened, and morphing geographies of production, distribution and consumption. Examining spatio-market practices offers a novel opportunity to discern the cultural, political and economic dimensions of this moment, everyday effects and implications.

In this regard and thinking more broadly, we believe that the concept can be used as a lens to reveal what it takes to realise markets, and what and whom such realisations serve. This possibly hinges on discarding any ‘unit-like’ treatment of space or spatial concepts and accepting that there are multiple market practices and multiple market spaces that are necessarily made and navigated in ways that are ‘real’ in their effects.

## Footnotes

1 We took a great deal of inspiration from Harvey (2006) and Schwanen (2019) when designing the table and inputting content. The former provides two tables in his seminal chapter–*Space as a Keyword* (Harvey, 2006). The latter presented a table similar to ours as part of a talk–*Thinking Space Relationally*–given during a British Sociological Association (BSA) workshop titled ‘Researching Relational Space: Concepts, Methods and Implications.’ We found Schwanen’s (2019) table particularly helpful when trying to read and make sense of Lefebvre and Harvey in conjunction. Please contact this paper’s first author for a copy of the slides.
